# Transient Pinning and Pulling: A Mechanism for Bending Microtubules

**DOI:** 10.1371/journal.pone.0151322

**Published:** 2016-03-14

**Authors:** Ian A. Kent, Parag S. Rane, Richard B. Dickinson, Anthony J. C. Ladd, Tanmay P. Lele

**Affiliations:** Department of Chemical Engineering, University of Florida, Gainesville, FL, United States of America; University of Illinois at Chicago, UNITED STATES

## Abstract

Microtubules have a persistence length of the order of millimeters *in vitro*, but inside cells they bend over length scales of microns. It has been proposed that polymerization forces bend microtubules in the vicinity of the cell boundary or other obstacles, yet bends develop even when microtubules are polymerizing freely, unaffected by obstacles and cell boundaries. How these bends are formed remains unclear. By tracking the motions of microtubules marked by photobleaching, we found that in LLC-PK1 epithelial cells local bends develop primarily by plus-end directed transport of portions of the microtubule contour towards stationary locations (termed pinning points) along the length of the microtubule. The pinning points were transient in nature, and their eventual release allowed the bends to relax. The directionality of the transport as well as the overall incidence of local bends decreased when dynein was inhibited, while myosin inhibition had no observable effect. This suggests that dynein generates a tangential force that bends microtubules against stationary pinning points. Simulations of microtubule motion and polymerization accounting for filament mechanics and dynein forces predict the development of bends of size and shape similar to those observed in cells. Furthermore, simulations show that dynein-generated bends at a pinning point near the plus end can cause a persistent rotation of the tip consistent with the observation that bend formation near the tip can change the direction of microtubule growth. Collectively, these results suggest a simple physical mechanism for the bending of growing microtubules by dynein forces accumulating at pinning points.

## Introduction

Microtubules play critical roles in cell functions such as mitosis, intracellular transport, and motility. They are the most rigid of the three cytoskeletal elements (microtubules, intermediate filaments, and F-actin [1]), and isolated microtubules are straight over length scales of millimeters [2]. Microtubules can buckle by polymerizing against obstacles [3–5], and their bent appearance in cells [6–8] is suggestive of a mechanical role in which they bear compressive loads [9, 10]. In this way, microtubules may help stabilize and maintain cell shape [11, 12].

However, microtubule bending does not only derive from polymerization against a barrier, because local bends also develop when the tips are polymerizing freely. By “freely polymerizing,” we mean that the microtubule tip moves to accommodate the additional length, which is distinct from the situation when the polymerizing tip is immobilized by the cell periphery or other obstacle. Moreover, polymerization forces cannot explain wavy microtubule growth from the centrosome, which is observed in tip-tracking experiments [13, 14]. Bicek et al. [15] have hypothesized that tangential forces generated by molecular motors can bend a microtubule by transporting portions of it (referred to as “segments” from now on) toward an immobile point on its contour. However, such pinning points have not been observed during the bending of freely growing microtubules, nor have the motors that might push the segments towards these pinning points been identified.

*In vitro* experiments with reconstituted microtubules have shown the feasibility of microtubule bend formation due to the activity of myosin motors [16]; retrograde flow of actomyosin can also cause microtubule buckling [17, 18]. However, Bicek et al. have argued against a role for actomyosin contraction in driving anterograde flow of microtubule bends, at least in LLC-PK1 epithelial cells [15]. Microtubule motors like kinesin [19] may drive microtubule bending but others have suggested that dynein is the dominant motor pulling along microtubule lengths [15, 20]. Thus the mechanisms for bend formation away from the cell periphery remain unclear.

Forces generated by dynein, kinesin, and actomyosin suggest different outcomes for the *direction* of microtubule-segment transport during the development of a bend. Dynein would be expected to translate segments from the minus end to the plus end, while kinesin would translate segments from the plus end to the minus end [15, 20]. Actomyosin contraction would bring in segments from both minus and plus ends. We hypothesized that determining the predominant direction of microtubule motion during bend formation, specifically the frequency of bends created by minus-end directed versus plus-end directed transport, could help identify the motors and mechanisms responsible for the shapes of microtubules in cells.

To detect the direction of transport of microtubule segments, photobleaching was used to create fiduciary markers along the microtubule length, which could then be used to follow individual segments as they reptate along the microtubule contour. We tracked the positions of these markers in LLCPK-1α cells, porcine kidney epithelial cells stably expressing GFP-α-tubulin. We observed that bends form primarily by plus-end directed transport of microtubule segments toward stationary segments along the microtubule. These pinning points were found to be transient, with a typical lifetime of less than 1 minute. When the pinning points released, the stationary segments started to move and the bends relaxed. Dynein inhibition eliminated the directional bias of microtubule transport and reduced the incidence of bend formation. We propose a model based on these observations, which involves dynein motor forces pushing segments towards a transient pinning point. Simulations of the development of microtubule bends correlate well with experimentally observed shapes and time scales. The model explains how bends forming near a growing microtubule tip can cause changes in direction of the growing microtubule.

## Results

### Microtubules bend against transiently immobilized segments

**Fig 1:**
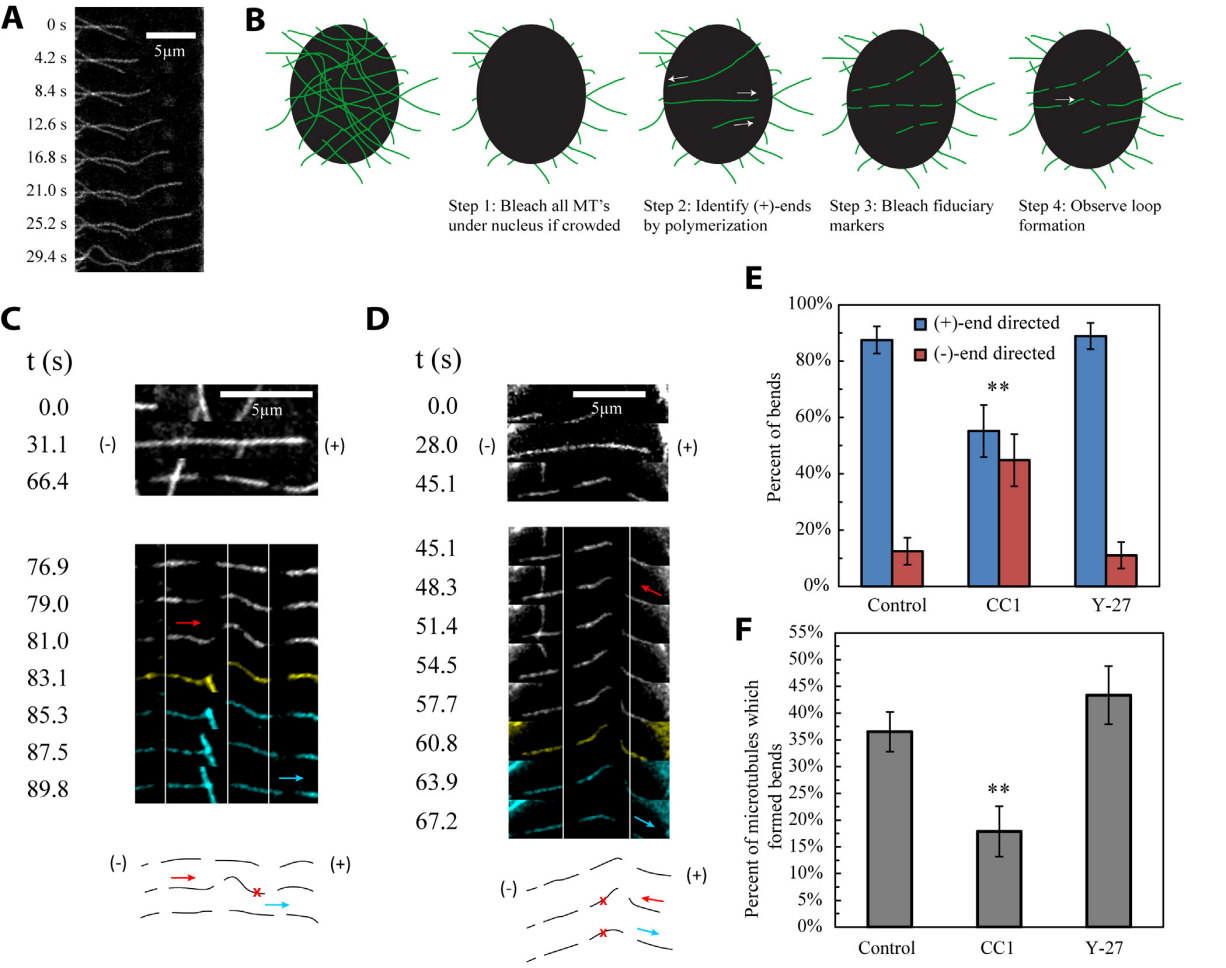
Local bends develop by dynein-dependent translation of microtubule segments from the minus end. (A) Image sequence shows bending of a freely growing microtubule, demonstrating that pinning of the plus end is not required for bends to develop. (B) Experimental Technique. Fluorescent microtubules under the cell nucleus are photobleached to allow for easier analysis of individual microtubules. Only newly polymerized segments of microtubules are fluorescent after bleaching. Microtubule plus ends are identified by their polymerization, since minus ends do not polymerize in cells. Once new microtubules grow to a sufficient length, fiduciary markers are bleached onto them so we may observe the lengthwise translation of different regions along their length. (C) The top panel shows a microtubule that polymerizes to the right over the course of 31 seconds and is bleached with a pattern of dashes at 66 seconds. A local bend then forms (white frames in bottom panel). During development of the bend, the minus end (left) side of the microtubule moves towards the plus end, and buckles against a stationary microtubule region, indicating pinning. The local bend then maintains its shape for some time (yellow frame) before relaxing. Bend relaxation (cyan frames) occurs by movement of the previously stationary right side toward the plus end, indicating unpinning of the microtubule, while the left side is stationary. Vertical white lines are provided to help visualize the movement of fiduciary markers. A cartoon trace of the microtubule’s shape evolution is provided below, in which the pinning point is marked with an “x” and translation of different regions of the microtubule during development and relaxation of the local bend is marked with red and cyan arrows, respectively. (D) The top panel shows that the microtubule polymerizes to the right over the course of 28 seconds and is then pattern bleached. A local bend develops by translation of the plus end (right) side of the microtubule toward the minus end, the opposite of what occurs in C. Also different from the microtubule in C, the bend relaxes away from the pinning point (in this case back toward the plus end), with the pinning point staying intact throughout. All colors and symbols are the same as in part C. (E) Plot showing the percentage of bends that formed by microtubule translation towards the plus and minus ends in control (n = 48 bends from 21 cells), dynein inhibited (CC1; n = 29 bends from 16 cells), and myosin-inhibited (Y-27; n = 45 bends from 18 cells) cells. Error bars indicate standard error. (F) Proportion of visible microtubules that bent in three minutes following photobleaching of the area under the nucleus in control (n = 167 microtubules from 9 cells), dynein inhibited (CC1; n = 67 microtubules from 6 cells), and myosin-inhibited (Y-27; n = 83 microtubules from 7 cells) cells. Error bars indicate standard error.

Microtubules commonly form local bends, even when the microtubule plus end is freely polymerizing (Fig 1A, S1A Movie). Thus polymerization against an obstruction such as the cell membrane is not necessary for formation of local bends. The bends have a characteristic “oxbow” shape in which the curvature changes sign twice. We observed the direction of tangential motion of microtubules during bend formation by photo-bleaching fiduciary markers onto microtubules under the nucleus. Before photobleaching fiduciary markers, cells were imaged to select growing microtubules. This procedure allowed for an unambiguous identification of the growing plus end, because free minus ends never polymerize in cells [6, 21–25]. The nuclear region was selected because it offered a dark background against which the microtubules were clearly visible and was far from the cell periphery (Materials and Methods and Fig 1B). During bend formation, the bleached microtubule segments were consistently observed to transport towards the bend from only one side (kymograph in Fig 1C, S1C Movie), while remaining stationary on the other side. Hence, translation of the microtubule towards a stationary region, or “pinning point,” appeared to be a consistent characteristic of local bend formation. After some time the bend relaxed, either through motion of the pinning point (Fig 1C) or motion of the bend while the pinning point itself remained stationary (Fig 1D, S1D Movie).

Having established that tangential motion toward stationary segments produces bends in microtubules, we counted the frequency of bends formed by translation toward the plus end and by translation toward the minus end. The majority of bends under the nucleus (87.5 ± 4.8% SE) were formed by translation of microtubule length from the minus end rather than from the plus end (Fig 1E). This argues against kinesin and myosin as dominant drivers of local bend formation in these cells and supports the hypothesis that dynein is involved in bend formation. It also implies that the additional microtubule length required to form bends does not come from plus-end polymerization.

### Dynein, but not myosin, is involved in formation of local bends under the nucleus

To determine whether dynein is involved in local bend formation under the nucleus, we inhibited dynein activity by over-expression of the fluorescently tagged CC1 domain of p150 (Glued), which is the dynein-binding domain of the dynactin complex [20, 26–30]. Inhibition of dynein activity was validated by dispersion of the Golgi apparatus (S1 Fig). In cells expressing DsRed-CC1, the percentage of bends caused by translation of the microtubule from the minus end was reduced significantly, to 55.2 ± 9.2% (χ^2^ = 10.16, p = 0.001; Fig 1E). Therefore, the bias in the direction of microtubule translation is due to dynein force generation. Inhibiting myosin activity by treating cells with Y-27632, a Rho kinase inhibitor [31], had no effect on the directional bias of microtubule translation (Fig 1E), in agreement with previously reported results in this cell type that argue against a role for actomyosin contraction in bend formation [15].

To determine the effect of dynein inhibition on the probability of bend formation, the area under the nucleus was bleached, and microtubules growing back into the area were observed over 3 minutes. New segments of microtubule that polymerized to a length of at least 5 µm under the nucleus over the observation period exhibited a reduced frequency of bend formation in dynein-inhibited cells compared to control cells, from 36.5 ± 3.7% in control to 17.9 ± 4.7% in CC1-expressing cells (χ^2^ = 7.72; p = 0.005; Fig 1F). This result adds support to the idea that dynein is acting to form bends from translation of microtubule segments from the minus end rather than to inhibit bend formation from translation of segments from the plus end. Inhibiting dynein not only equalized the probability of bending in the anterograde and retrograde directions, but also decreased the overall probability that a microtubule will bend. On the other hand, treating cells with Y-27 to disrupt myosin contractility did not decrease the frequency of bend formation, consistent with myosin activity not being a major cause of local bending (Fig 1F).

### A mechanism for microtubule bend formation by dynein-mediated transport towards a pinning point

**Fig 2:**
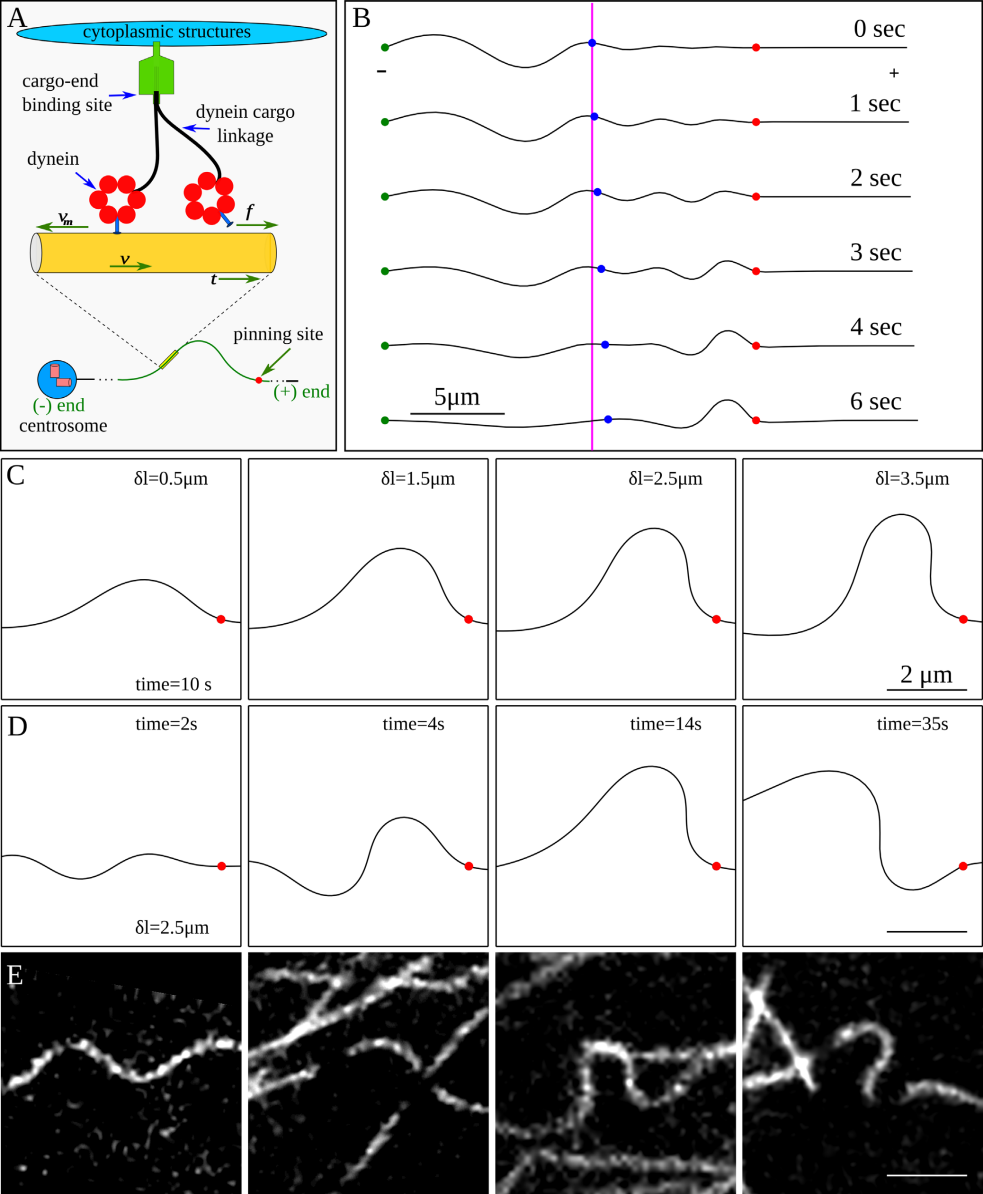
Simulations of dynein mediated bend formation in microtubules. (A) The cartoon illustrates a model for the dynein-generated force on a microtubule. A net tangential force is experienced by the microtubule due to collective motor activity. (B) Simulated bend development by tangential forces using the model represented in A. The red dot is the location of the pinning site, the green dot indicates the minus end of the microtubule, and the blue dot is a point that marks the beginning of the microtubule segment that would be observed in a typical experiment. The magenta colored line marks initial position of the blue dot. The figure shows microtubule translation due to cytoskeletal dynein motors. The excess length behind the blue marker translates into the viewing window by motor activity, leading to a pronounced bend on the minus side of the pinning point. The excess length is equal to the maximum displacement of the blue marker from its original position. (C) Simulated shapes from varying excess lengths, generated after 10 seconds. (D) Snapshots of a segment of a microtubule at different times, showing how different shapes can develop if the microtubule is pinned for a long time (excess length of 2.5 μm). (E) Microtubule shapes observed in living cells.

We applied a mathematical model [20, 32] for dynein force generation on microtubules (Fig 2A) to explain bend formation near a pinning point. In this model, an ensemble of dynein motors linking the microtubule to the surrounding cytoplasmic structures [20] or to the nucleus [32] exert a tangential force directed towards the plus end of the microtubule. The microtubule is modeled as an elastic filament subjected to tangential pulling forces from the dynein motors, and a lateral viscous force due to protein friction arising from transient dynein linkages [20].

Fig 2B shows results of a simulation in which the microtubule is pinned at the minus end (green circle) and also pinned (red circle) at a significant distance (8 microns) from the free plus end. This configuration represents a centrosome-bound microtubule with a (transient) pinning point at a distance from the growing tip. The microtubule to the right of the purple line represents the region lying within the field of view of the microscope. We assume there must be sufficient curvature to provide the necessary excess length (the difference between the contour length of the microtubule and the linear distance between the endpoints) to form a bend, since the minus end is anchored at the centrosome and microtubules are essentially inextensible.

Initially (t = 0), the microtubule in view appears straight, but as tangential dynein forces drive the excess length into the region of view, a bend begins to form near the pinning point (Fig 2B). Using the estimated motor friction for nuclear-bound dynein [32], simulations predict that bends are formed in about 5–10 s, which is consistent with the timescales of the experimental observations.

Simulations support the conclusion that bends are formed when the transport of excess length by dynein from the minus end direction is halted by stationary (pinned) segments. The resulting bend shapes depend on the amount of excess length, but are insensitive to the shape of the microtubule segments acting as the source of excess length (i.e. the region to the left of the field of view in Fig 2B). Typical microtubule bends arising from various excess lengths are shown in Fig 2C; these are similar to shapes observed experimentally (Fig 2E).

Microtubule bends formed in the simulations are not static but undergo limit cycles consisting of slow oscillations between different bend orientations (Fig 2D). The cycle time varies inversely with excess length; from tens of seconds for large excess lengths (> 3 microns) to several minutes for small excess lengths (< 1 micron). Although bends in experiments were sometimes observed to change orientation (S2 Fig), we did not observe limit cycles with multiple periods because pinning points were too short-lived (Fig 1C and 1D), typically existing for 20–40 seconds and only occasionally for as long as 1 minute.

### Bending near the microtubule plus end tip leads to tip rotation

**Fig 3:**
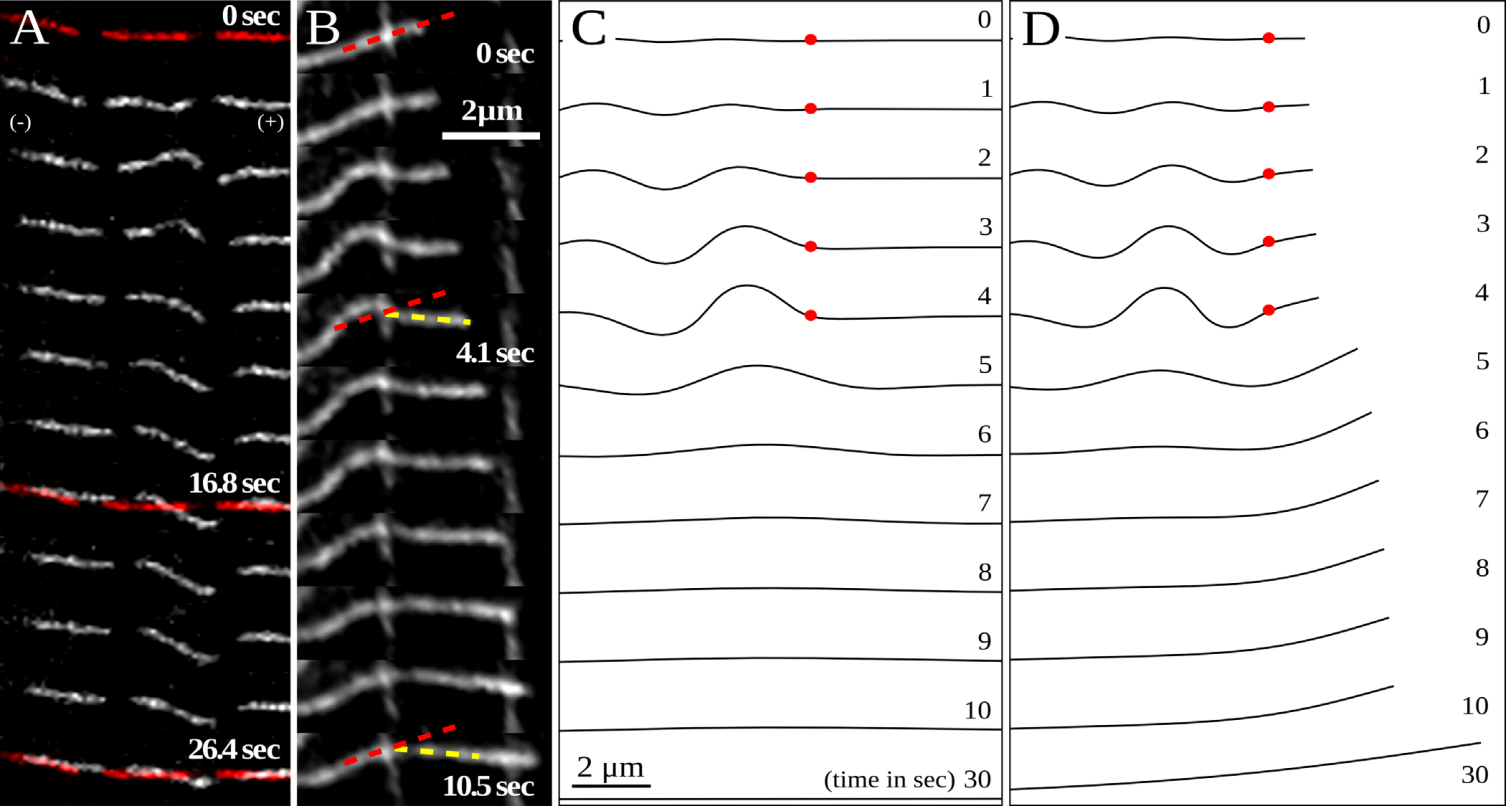
Rotation of the microtubule tip due to local bend formation. (A) Images show that the microtubule shape on either side of the bend remained almost the same, and the initial shape was ultimately restored after the bend relaxed (initial and final shapes marked in red). (B) An example of a microtubule that changes direction of polymerization due to a local bend near the tip. Initially the filament is aligned at an angle of roughly 30 degrees to the horizontal (marked by red dash), but as soon as the bend starts to form, the tip starts to rotate toward the horizontal direction. The plus end continues to grow as the bend develops over the course of about three seconds. The bend relaxes after about 10 seconds, but the tip keeps growing in the newly acquired direction (marked in yellow). (C) Simulation of local bend formation when the pinning point is far from the tip (as in the experiment shown in panel A). The red dot indicates the pinning site which is initially at a distance of 8 μm from the tip. A bend forms within the first 5 seconds, after which the pinning point is released. The segment on the plus side of the pinning point does not rotate because of the large lateral friction along its length. As a result, the direction of microtubule growth remains unchanged. (D) Local bend formation when the pinning point is close (0.5 μm) to the tip (as in the experiment shown in panel B). The short microtubule segment to the plus side of the pinning point changes direction during bend formation, but microtubule growth during the pinning event increases the lateral friction and prevents the tip relaxing back to its original direction after the pinning point is released. There is a net change in the tip direction even after 30 seconds.

When local bends form far from the microtubule tip, the shape changes did not propagate very far from the bend. For example, Fig 3A shows a typical experiment when a bend formed far from the tip. The bend formed and relaxed over a period of about 25 s, but the microtubule shape and position on either side of the bend remained nearly the same, and the initial shape was restored after the bend relaxed. However, when a bend formed near the tip of a growing microtubule (Fig 3B), the direction of polymerization changed and the new direction persisted even after the bend relaxed.

**Fig 4:**
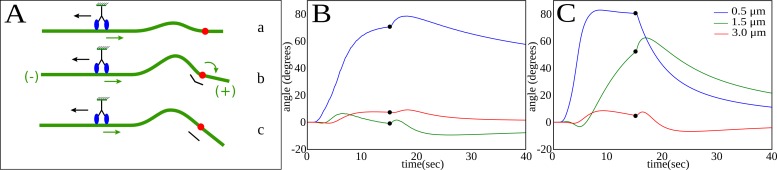
Effect of overhanging segment length on the rotation of the tip. (A) The sketch shows how a local increase in curvature during bend formation drives tip rotation. A local bend (a) generates a bending moment across the pinning point (b). Motion of the overhanging segment is opposed by friction, but given enough time, the tip rotates to straighten the segment (c). (B) Orientation of a growing microtubule tip as a function of time. The microtubule is initially pinned at different distances from the tip: 0.5 micron (blue), 1.5 micron (green) and 2.5 micron (red) and unpinned after 15 seconds (black dot). (C) Orientation of a non-polymerizing microtubule tip as a function of time. The microtubule is initially pinned at different distances from the tip: 0.5 micron (blue), 1.5 micron (green) and 2.5 micron (red) and unpinned after 15 seconds (black dot).

Bend formation generates a bending moment at the pinning point, as illustrated in Fig 4A. When the segment on the plus-end side of the pinning point is sufficiently short, the resulting moment causes the tip to rotate to a new direction for subsequent growth. However, when the segment is long, the large lateral friction prevents rotation and the initial orientation is preserved. These qualitative insights are confirmed by numerical simulations, illustrated in Fig 3C and 3D, where the motion of growing microtubules with either a long or a short segment past the pinning point were tracked through a cycle of pinning and release. The time evolution of the microtubule shape and tip orientation closely resembles the corresponding dynamics observed experimentally. The microtubule pinned far from the tip (Fig 3C) continues growing in the same direction after release, whereas the one pinned near the tip changes its direction of growth. Thus, our model for dynein force generation accounts for the observed correlation between the formation of bends and a sharp change in the direction of a growing microtubule tip; furthermore it explains why the bend must be close to the growing tip in order to cause a change of direction.

We repeated the simulations shown in Fig 3C and 3D with different segment lengths between the pinning point and the tip, which we refer to as the overhang. The resulting tip orientations are plotted versus time in Fig 4B. When the distance between the pinning point and the tip is small (blue line) the tip rotates rapidly as the bend forms. However, after pinning, the overhang acquires additional frictional resistance because of polymerization, which opposes the relaxation in orientation after the pinning point is released. This leads to a substantial rotation of the tip, even 25 s or more after the pinning point is released, but when the pinning is more than about 1 μm from the microtubule tip (green and red lines), there is no significant change of direction.

When the overhang is small (<1 μm), microtubule polymerization contributes significantly to the net rotation of the tip. In Fig 4C, the rotation of the tip of non-polymerizing microtubules is shown. Here the short (0.5 μm) overhang rotates rapidly after pinning, but when the bend relaxes it tends to return to its initial direction because the friction remains small. The longer (1.5 μm) overhang now rotates significantly (because its friction remains constant during bend formation) but it also relaxes once the pinning point it released, although more slowly than the 0.5 μm overhang. The longest overhang has sufficient friction that it scarcely rotates, even when the tip is not growing.

## Discussion

Polymerization forces from a growing microtubule can cause compressive buckling if the tip is pinned at the cell periphery, but it is difficult to see how this mechanism can generate local bends far from the tip or how they can propagate back from the periphery. In addition, such a model cannot explain how local bends form when the microtubule tips are free. However, tangential force generation by molecular motors can transport curved segments towards any stationary point (including the cell periphery). In this paper we focused on bends that develop under the nucleus, where individual microtubules could be easily seen and where they were far from the cell periphery. Tracking of fiduciary markers along the microtubule length showed that transport of length during the formation of a bend occurs primarily from the minus-end direction; the bend develops because excess length is transported toward pinned microtubule segments; and dynein activity increases the frequency of bend formation and helps drive excess length preferentially from the minus end. Simulations that account for dynein activity and microtubule mechanics correctly predict the observed shapes and time scales of bend formation, supporting our physical explanation for how dynein generates local bends in microtubules. Dynein activity also explains previously reported transport of microtubule bends toward the cell periphery [15].

While bends occur primarily by translation of microtubule segments from the minus end direction, in a small but significant fraction of the observed bend formations, the bends developed by transport from the plus end direction. In dynein-inhibited cells, the overall frequency of bending decreased and the probability of bending in either direction was equalized. This suggests that other mechanisms exist by which microtubule bends can form, potentially including kinesin motoring [19] and actomyosin contraction [3, 16]. While we do not rule out these other mechanisms contributing to the bending, the primary mechanism in LLC-PK1 cells appears to be dynein mediated forces.

Our simulations show that tangential forces pushing mobile microtubule segments against stationary pinning points are mechanically sufficient to give rise to the bends observed in experiments. The time scale of bend formation depends on the lateral friction from transient motor linkages. The fact that the timescales of bend formation can be predicted with the same motor parameters that were estimated from a separate study on nuclear rotation [32] increases our confidence in the proposed model.

Microtubules in cells do not grow in straight lines [13]. In previous experiments, we have shown that the waviness in microtubule growth is primarily due to dynein activity [14]. Tip rotation due to bend formation may be a means whereby a growing microtubule amplifies existing excess length through a succession of pinning and unpinning events. A pinning event near the tip, together with bend formation, causes a change in the direction of growth and therefore generates additional excess length because the microtubule is following a wavier path. This would appear as random fluctuations in a tip-tracking experiment, because the developing bend behind the tip remains unobserved. We note that this mechanism requires preexisting excess length, but its origin remains unclear because microtubules can grow along wavy paths even when dynein, myosin, or kinesin are inhibited [14].

In summary, the experimental results and simulations presented here support the existence of two related mechanisms of microtubule bending by dynein: bend formation near pinning points and resulting reorientation of the direction of polymerization. These findings continue to point to the critical role of motor forces and protein friction in governing microtubule bending dynamics *in vivo*.

## Materials and Methods

### Cell culture

LLCPK-1α cells [33], a porcine kidney epithelial cell line stably expressing GFP-α tubulin, were cultured in Opti-MEM (Gibco, Grand Island, NY) supplemented with 10% Donor Bovine Serum (Gibco). For imaging, the cells were cultured on glass-bottom dishes (WPI) that had been coated with 5 μg/mL fibronectin (BD Biocoat, Franklin Lakes, NJ) overnight at 4°C.

Cells were transfected using Lipofectamine 2000 (Life Technologies, Carlsbad, CA). The plasmid dsRed-CC1 [26] (gift from Dr. Trina A. Schroer, Johns Hopkins University) was used in this study. Myosin activity was inhibited by treating cells with ROCK-inhibitor Y-27632 (EMD Millipore, Billerica, MA) at a concentration of 25 μM for one hour prior to imaging.

### Imaging

Imaging was performed on a Nikon TE2000 microscope with a Nikon A1 confocal laser unit. During microscopy, cells were maintained in an environmental chamber in which the temperature was kept at 37°C, the CO_2_ level at 5%, and the relative humidity at 100%. Depending on the density of microtubules under the nucleus at the start of imaging, the entire area under the nucleus was bleached to allow for easy identification of individual microtubules. After bleaching, only newly polymerized microtubule segments were fluorescent in the bleached area. Once fluorescent microtubule segments grew back into the sub-nuclear region, dashes were bleached into the microtubules, after which a time lapse image sequence was taken to capture bending events. All imaging of microtubules was performed with a 488nm wavelength laser at a power of 0.69% and a 60x oil immersion objective, with images being taken at one second intervals. Bleaching was performed at 35% laser power for one second.

### Analysis

To quantify the number of bends produced by microtubule length translation from the plus end or minus end directions, time lapse videos were visually analyzed. Brightness and contrast were adjusted, and bilinear interpolation and the ImageJ Image Stabilizer plugin (Li K, Carnegie Mellon Univ 2008) were used to ease visualization using ImageJ (NIH). Bends which developed in dashed microtubules were categorized as being formed by translation toward the plus or minus end. If a bend was too small to see any translation in the surrounding dashes, it was disregarded.

For quantifying the frequency of bend formation under the nucleus, the sub-nuclear area was bleached and imaged for 3 minutes. The number of newly polymerized microtubule segments reaching a length of at least 5 μm over the observation period was counted, as well as whether a bend formed in each of them.

Statistical analysis was performed by Pearson chi-square analysis between each condition and the control and then determining the corresponding p-value for one degree of freedom.

### Model for dynein forces

Motor forces were included as linkages distributed along the length of the microtubule, as illustrated in Fig 2A. We imagine that a particular segment of the microtubule is captured by a cytoskeletal-bound dynein motor and bound for times of the order of $k_{off}^{-1}$. On average, the stochastic binding and unbinding of molecular motors acts as a frictional force, which is combined with the tangential pulling force of the walking motors. Based on these ideas, an expression for the average motor force can be derived (Supporting Information in [20])

$$\vec{K}=f_{max}\left(1-\frac{\vec{v}\cdot \vec{t}}{v_{0}}\right)\vec{t}-\gamma \vec{v}\cdot \left(\overset{↔}{1}-\vec{t}\vec{t}\right)$$ (1)

where $\vec{v}$ is the velocity of the segment the motor is bound to, $\vec{t}$ is the local tangent to the microtubule contour, and $\overset{↔}{1}$ denotes the unit tensor. There are three parameters in this model (Table 1): the maximum pulling (or stall) force of the motor *f*_*max*_, the velocity of a force-free dynein motor walking along the microtubule *v*_0_, and the lateral friction from motor binding and unbinding *γ*. Two of these parameters have established values in the literature: *f*_*max*_ = 8 pN and *υ*_0_ = 0.8 *μ*m⋅s^−1^ [34, 35]. The motor friction for nuclear-bound dynein motors, *γ* = 56 pN⋅s⋅*μ*m^−1^ was determined by fitting to data on nuclear rotation rates [32].

**Table 1:** Model Parameters.

Symbol	Parameter	Range	Source	Value Used
*f*_*max*_	Maximum dynein force	5–8 pN	[34]	8 pN
*v*_*0*_	Dynein speed (no force)	0.8 μm∙s^-1^	[35]	0.8 μm∙s^-1^
*γ*	Lateral friction coeff.	20–1000 pN∙s∙μm^-1^	[32]	56 pN∙s∙μm^-1^
*ρ*	Dynein density (#/length)	2 μm^-1^	[20]	2 μm^-1^
*v*_*pol*_	MT polymerization speed	0.1–0.2 μm∙s^-1^	[36, 37]	0.1 μm∙s^-1^

### Simulation methods

Microtubule dynamics was simulated by incorporating the model for dynein forces with a standard model for the bending of an elastic filament [14, 38]. The microtubule contour is represented by a set of discrete segments of length *h*, and the strain energy in a bent microtubule of length *L* = *Nh* (free from external couples) is approximated by a sum of the bending energies of a finite number of joints,

$$H=\frac{Bh}{2}\sum_{m=1}^{N-1}C_{m}^{2}$$ (2)

The curvature *C*_*m*_ is related to the angle between adjacent segments, and is given by the formula $C_{m}^{2}=2\left(h^{2}-{\vec{r}}_{m,m-1}\cdot {\vec{r}}_{m+1,m}\right)/h^{4}$, where ${\vec{r}}_{m,m-1}$ is between nodes m and m-1. The discrete approximation to *H* can then be differentiated to find the elastic force on each node representing the microtubule contour. In addition to the elastic forces, there is also a constraint force that is required to maintain the length of the individual segments, ${\vec{r}}_{m,m-1}\cdot {\vec{r}}_{m,m-1}=h^{2}$.

In the over-damped limit, the nodal velocities ${\vec{v}}_{m}$ are obtained from the balance between elastic and motor forces:

$$-\frac{\partial H}{\partial {\vec{r}}_{m}}+nh{\vec{K}}_{m}=0,$$ (3)

where ${\vec{K}}_{m}$ is the motor force on node *m*, and *n* is the density of cytoskeletal-bound dynein motors; as in previous work [14, 20], we take *n* = 2 μm^-1^.

## Supporting Information

S1 Fig Altered Golgi organization in LLCPK-1α cells when expressing dsRed-CC1. Immunostaining against 58K Golgi Marker was used to visualize the Golgi. (A) Cells not expressing dsRed-CC1. There is a compact Golgi apparatus adjacent to the nucleus in both cells (white arrows), and Golgi vesicles away from the nucleus have an elongated shape. (B) Cells expressing dsRed-CC1. There is no compact Golgi region near the nucleus, and Golgi vesicles throughout the cell are disorganized compared to the cells in A.
(TIF)

S2 Fig Flipping of a local bend at the minus end of a free microtubule. (TIF)

S1 Movie (A) Movie of a microtubule forming local bends as it polymerizes. Corresponds to Fig 1A. (C) Movie of local bend formation by microtubule length translation from the minus end and relaxation by movement of the pinning point. Corresponds to Fig 1C. (D) Movie of local bend formation by microtubule length translation from the plus end and relaxation by translation away from the pinning point (back toward the plus end). Corresponds to Fig 1D.
(ZIP)
