# Supplementary figures and images for: Transient Pinning and Pulling: A Mechanism for Bending Microtubules
Source: PLoS One. 2016 Mar 14;11(3):e0151322. doi: 10.1371/journal.pone.0151322 (PMC4790857; doi:10.1371/journal.pone.0151322)

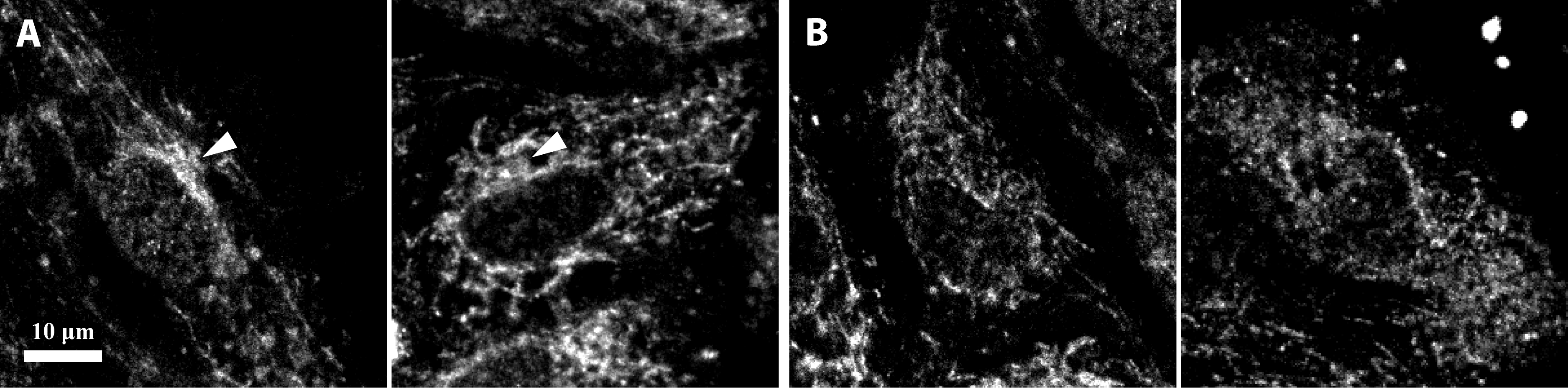

Supplement: S1 Fig — Immunostaining against 58K Golgi Marker was used to visualize the Golgi. (A) Cells not expressing dsRed-CC1. There is a compact Golgi apparatus adjacent to the nucleus in both cells (white arrows), and Golgi vesicles away from the nucleus have an elongated shape. (B) Cells expressing dsRed-CC1. There is no compact Golgi region near the nucleus, and Golgi vesicles throughout the cell are disorganized compared to the cells in A. (TIF) [file pone.0151322.s001.tif]

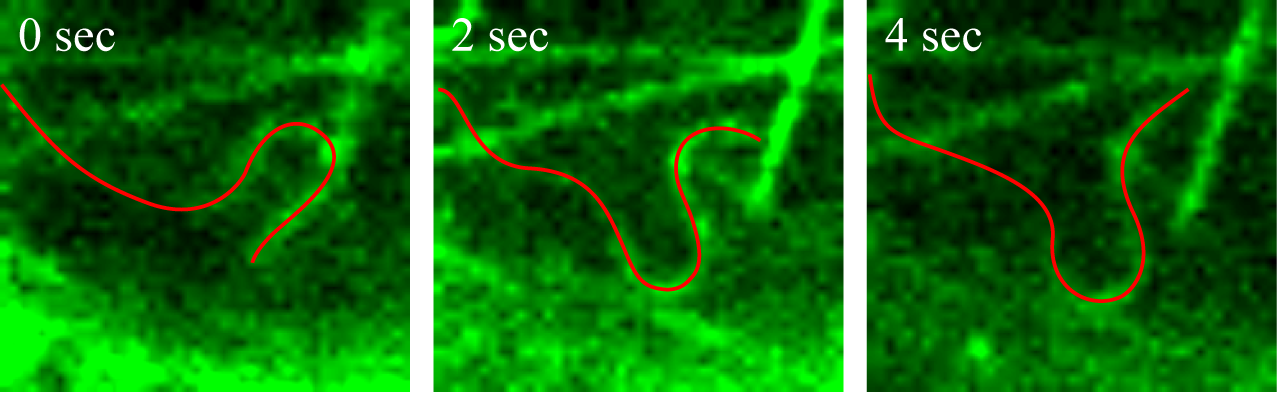

Supplement: S2 Fig — (TIF) [file pone.0151322.s002.tif]
